# Intestinal microbiota changes in Graves’ disease: a prospective clinical study

**DOI:** 10.1042/BSR20191242

**Published:** 2020-09-04

**Authors:** Hui-xian Yan, Wen-cheng An, Fang Chen, Bo An, Yue Pan, Jing Jin, Xue-pei Xia, Zhi-jun Cui, Lin Jiang, Shu-jing Zhou, Hong-xin Jin, Xiao-hong Ou, Wei Huang, Tian-pei Hong, Zhao-hui Lyu

**Affiliations:** 1 Department of Endocrinology, Beijing Haidian Hospital, Beijing Haidian Section of Peking University Third Hospital, Beijing 100080, China; 2 Department of Clinical Laboratory, Beijing Haidian Hospital, Beijing Haidian Section of Peking University Third Hospital, Beijing 100080, China; 3 Department of Endocrinology, Peking University Third Hospital, Beijing 100191, China; 4 The Department and Key Laboratory of Endocrinology and Metabolism, PLA General Hospital, Beijing 100853, China

**Keywords:** Case-control, Grave's disease (GD), Hyperthyroidism, Intestinal microbiota, Prospective, Thyroid-stimulating antibodies (TSAb)

## Abstract

Graves’ disease (GD) occurs due to an autoimmune dysfunction of thyroid gland cells, leading to manifestations consistent with hyperthyroidism. Various studies have confirmed the link between autoimmune conditions and changes in the composition of intestinal microbial organisms. However, few studies have assessed the relationship between the GD and the changes in intestinal microbiota. Therefore, the present study aimed to investigate changes in intestinal flora that may occur in the setting of GD. Thirty-nine patients with GD and 17 healthy controls were enrolled for fecal sample collection. 16S rRNA sequencing was used to analyze the diversity and composition of the intestinal microbiota. High-throughput sequencing of 16S rRNA genes of intestinal flora was performed on Illumina Hiseq2500 platform. Comparing to healthy individuals, the number of *Bacilli, Lactobacillales, Prevotella, Megamonas* and *Veillonella* strains were increased, whereas the number of *Ruminococcus, Rikenellaceae* and *Alistipes* strains were decreased among patients with GD. Furthermore, patients with GD showed a decrease in intestinal microbial diversity. Therefore, it indicates that the diversity of microbial strains is significantly reduced in GD patients, and patients with GD will undergo significant changes in intestinal microbiota, by comparing the intestinal flora of GD and healthy controls. These conclusions are expected to provide a preliminary reference for further researches on the interaction mechanism between intestinal flora and GD.

## Introduction

Graves’ disease (GD) is an autoimmune disease involving thyroid gland. Patients with GD present with a series of symptoms, including hyperthyroidism, heat intolerance, and weight loss [1,2]. It is generally believed that GD is caused by complex interactions involving genetic and environmental factors although the underlying reasons for the pathogenesis of GD have not been fully elucidated [3,4]. The functional interactions between thyroid gland and gastrointestinal system has been clearly verified [5,6]. The development and differentiation of intestinal mucosa cells is regulated by tri-iodothyronine (T3) [7]. Some variations in the intestinal microbiota can also indirectly affect thyroid function [8].

The intestinal microbiota constitutes approximately two-thirds of the total human microbiome. These microorganisms play active roles in promoting human health, such as modulating immune development, digesting dietary nutrients and maintaining metabolic homeostasis. Previous studies have confirmed that the disruption of the normal composition in intestinal flora is implicated in different disease conditions, such as inflammatory bowel disease, obesity and type 2 diabetes mellitus [9]. Some autoimmune diseases, type 1 diabetes mellitus [10], rheumatoid arthritis [11], multiple sclerosis [12] can cause a dysbiosis of intestinal microbiota. Hypothetically, thyroid autoimmune disorders would also interfere with normal homeostasis in intestinal microbiota.

The regulation of thyroid hormone metabolism by intestinal microbiota may be achieved through inhibiting deiodination and glucuronidation activities [13,14]. Intestinal submucosal immune cells exposed to unrecognized antigens will provoke autoimmune disorders [15]. Morphological and functional damages to the intestinal barrier have been reported in patients with autoimmune thyroiditis, and ultrastructural changes observed in the enterocytes of subjects with Hashimoto’s thyroiditis (HT) [8,16]. However, the effects and mechanisms of thyroid dysfunctions on microbiota have not been fully elucidated. Therefore, the purpose of the present study was to investigate possible changes in the composition of the intestinal microbiota of patients with GD through comparison with a group of normal population.

## Materials and methods

### Study design and setting participants

We conducted a cross-sectional study involving 39 GD patients and 17 healthy controls from April 2017 to December 2017. The healthy volunteers serving as controls were matched with the GD group in terms of age, sex, and body mass index (BMI). All patients with GD were recruited from the Outpatient Department of Endocrinology of Haidian Hospital, Beijing, China. The diagnosis of GD was made by an endocrinologist based on patient history, physical examination, ultrasound examination, and biochemistry. The inclusion criteria for the group comprising GD patients were: being diagnosed with GD according to the guidelines of Chinese Society of Endocrinology at the Beijing Haidian Hospital, Beijing Haidian Section of Peking University Third Hospital, Department of Endocrinology; not having used medicine for the treatment of GD for the last 6 months; a diagnosis of GD based on clinical manifestations of hyperthyroidism (history of marked weight loss, presence of diffuse goiter, presence of changes in the skin or nails), serum thyroid hormone levels and the titers of TSHR antibodies; having serum T3 levels higher than 2.44 nmol/l (normal range: 0.35–4.94 μIU/ml), T4 levels higher than 150.84 nmol/l (normal range: 62.68–150.84 nmol/l), thyroid stimulating hormone (TSH) less than 0.35 μIU/ml (normal range: 0.35–4.94 μIU/ml), and TSH receptor antibodies (TRAbs) higher than 1.75 IU/l (normal range: <1.75); a lack of history with gastrointestinal diseases in both patients with GD and normal healthy subjects; a lack of history in the use of prebiotics and antibiotics, 2 months prior to the study. The exclusion criteria were as follows: pregnancy; cigarette smoking; alcohol addiction; and recent (<3 months) use of antibiotics, hormonal medication or Chinese herbal medicine.

The protocols of the present study were approved by the Ethics Committee of the Beijing Haidian Hospital (No. 2017013). All subjects were informed of the nature of the study and they provided written informed consent. The procedures were conducted in accordance with approved guidelines.

### Samples collection

Fecal samples collected from 39 GD patients (11 males and 28 females) aged 15–67 years and 17 healthy subjects (6 males and 11 females) matched by age, were preserved at −80°C. All the samples from GD patients were collected at first diagnosis and hadnot been treated for GD before. Similarly, blood samples were collected from all subjects and stored at 4°C pending laboratory analysis.

### Genomic extraction and purification

After the initial treatment of the intestinal flora by homogenization, the genomic DNA of the gut microbiota from fecal samples was extracted using QIAamp Fast DNA Stool Mini Kit (QIAGEN China (Shanghai) Co., Ltd.). The quality and integrity of the extraction were evaluated by micro spectrophotometer and electrophoresis. Multiprimer amplification was performed using multi-PCR and touchdown-PCR with high-fidelity low-cycle PCR system using the extracted metagenomics DNA as template.

### Database construction and sequencing

Qualified libraries were sequenced using the Illumina Hiseq 2500 high-throughput sequencing platform. The PE read out obtained by HiSeq sequencing was spliced into a target region sequence according to the overlap relationship, and the target sequence subjected to quality control filtering. The filtered sequence was then compared with the reference database, and the chimeric sequence removed [17,18]. Finally, the sequence underwent optimization.

### Operational taxonomic unit cluster and species annotation

Operational Taxonomic Units (OTUs) cluster analysis was performed using the Uclust method in the QIIME software package based on 97% sequence similarity. Then, based on the Silva (Release128 https://www.arb-silva.de/documentation/release-128/) reference database, the RTP Classifier software (confidence cut-off = 0.8) was used to classify the OTUs of each sample.

### Statistical methods

Clinical parameters were analyzed by Statistical Package for the Social Sciences (SPSS), version 19.0 (SPSS Inc., 2010 Chicago, IL, U.S.A.) and *P*-value <0.05 was considered significant.

First, α-diversity analysis of all samples, including Observed species, Chao1, Shannon and Simpson, were performed with the intention of evaluating the richness and evenness of the samples. Second, β-diversity evaluations were further conducted between the two groups, including principal coordinate analysis (PCoA), principal component analysis (PCA) and non-metric multidimensional scaling (NMDS), with the motive of investigating differences between the group with patients with GD and the healthy control group [19]. Third, if these methods did not show significant indicators of differences between groups, it can be solved by ANOSIM analysis. Moreover, Partial Least Squares Discriminant Analysis (PLS-DA) was used to verify the difference between groups when the difference between groups is small, the sample size difference between groups is large, the group with a large sample size will play a leading role. Based on PLS-DA results, a linear discriminant analysis (LDA) Effect Size (LEfSe) comparison was applied, using non-parametric factorial Kruskal−Wallis (KW) sum-rank test. Finally, parameters with an LDA score greater than 2.0 were selected as the potential biomarkers between the two groups.

## Results

### Study population

All fecal samples of study participants were sequenced and analyzed. The demographics and clinical parameters of the subjects are as summarized in Table 1. Of all the 39 patients with GD, the majority (28, 72%) was female and the age ranged from 15 to 67 years. Among the 17 matched healthy controls, the majority were also female (11, 65%) and the age ranged from 13 to 62 years.

**Table 1:** Clinical and demographic features of GD patients and healthy controls (mean **±** SD)

Parameter	GD patients (*n*=39)	Healthy controls (*n*=17)	*P*-value
Gender (M/F)	11/28	6/11	0.671
Age (years)	37.49 **±** 12.95	33.42 ± 9.13	0.524
TT3 (nmol/l)	6.07 **±** 3.77	1.55 ± 0.13	*P*<0.01
TT4 (nmol/l)	219.48 **±** 74.99	76.9 ± 8.75	*P*<0.01
TSH (μIU/ml)	0.00 **±** 0.001	1.31 ± 0.71	*P*<0.01
TG-Ab (IU/ml)	268.58 **±** 336.61	9.68 ± 29.81	*P*<0.01
TPO-Ab (IU/ml)	422.09 **±** 420.23	0.75 ± 1.60	*P*<0.01
TRAb (IU/l)	15.76 **±** 12.77	<0.3	*P*<0.01

Abbreviations: TG-Ab, thyroglobulin antibody (0–4.11 IU/ml); TPO-Ab, thyroperoxidase antibody (0–5.61 IU/ml); TT4, total thyroxine (62.68–150.84 nmol/l); TRAb, thyrotropin receptor antibody (0–1.75 IU/l); TSH, thyroid-stimulating hormone (0.35–4.94 μIU/ml); TT3, total triiodothyronine (0.89–2.44 nmol/l).

### Gut microbiota of GD patients and healthy controls

**Figure 1:**
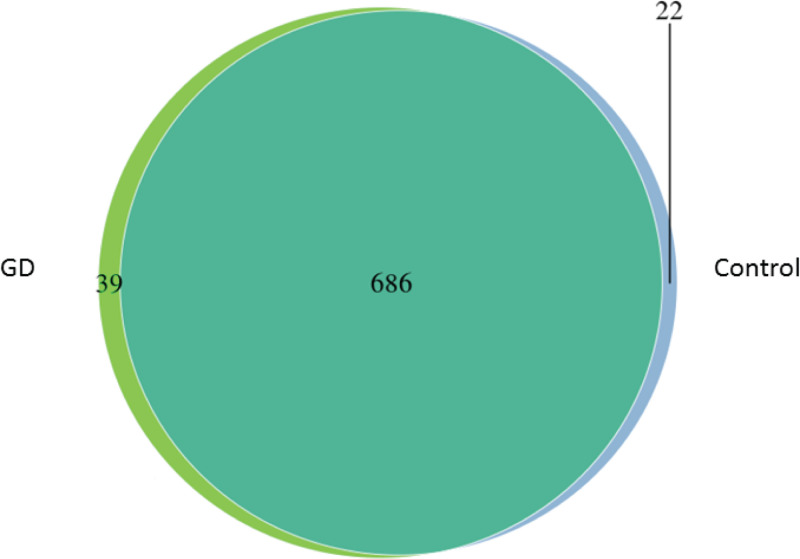
Venn diagram showing microbiota diversity between GD patients and healthy controls Overlap of OTUs in microbiota between the two groups is evident. Control, healthy controls; GD, GD patients.

To compare the intestinal microbiota between GD patients and healthy controls, the fecal samples were subjected to HiSeq sequencing. The microbiota diversity between the two groups was displayed by a Venn diagram (Figure 1). Significant differences in the microbial composition between the patients with GD and healthy cohorts were apparent (OTU similarity level ≥ 97%). As depicted in the Venn diagram, 686 of the 747 OTUs accounting for the total number of microbial species were common to all fecal samples. The number of species unique to the patients with GD and healthy groups were 39 and 22, respectively.

**Figure 2:**
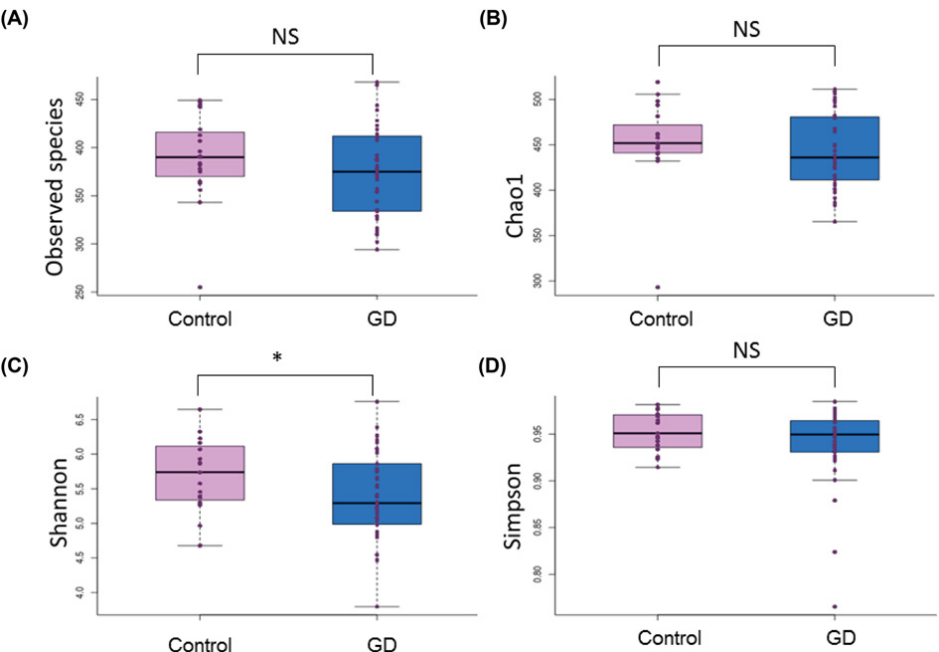
Microbiota diversity between GD patients and healthy controls Displayed using: (**A**) Observed Species, (**B**) Chao1, (**C**) Shannon, and (**D**) Simpson Diversity Indices. Control, healthy controls; GD, GD patients. *, significant difference.

The diversity of the samples within each group was further analyzed using the Observed Species, Chao1, Shannon, and Simpson Diversity Indices (Figure 2). The Chao1 index, which considers species richness, demonstrated that there was lower richness in the intestinal microbiota of GD patients compared with that of healthy controls (*P*=0.077). The same profile was observed with the Shannon diversity index which, additionally, revealed less evenness in the microbiota of GD patients compared with the healthy controls (*P*=0.025). Furthermore, a significant decrease in the mean diversity of microbial species was observed in the GD patient cohort when compared with the healthy controls.

**Figure 3:**
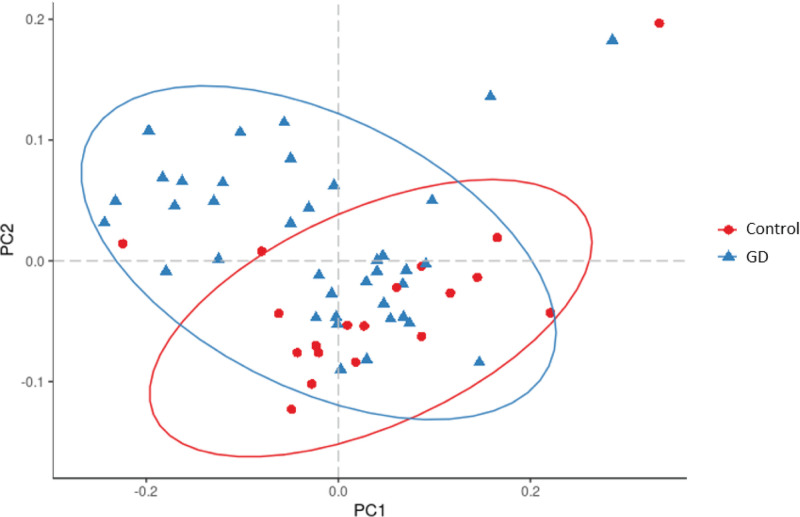
A PCoA plot based on the Bray–Curtis distance matrix showing microbiota diversity between GD patients and healthy controls The first two coordinates are plotted with the percentage of variability indicated on the axis. Each point represents a sample. Control, healthy controls; GD, GD patients.

**Figure 4:**
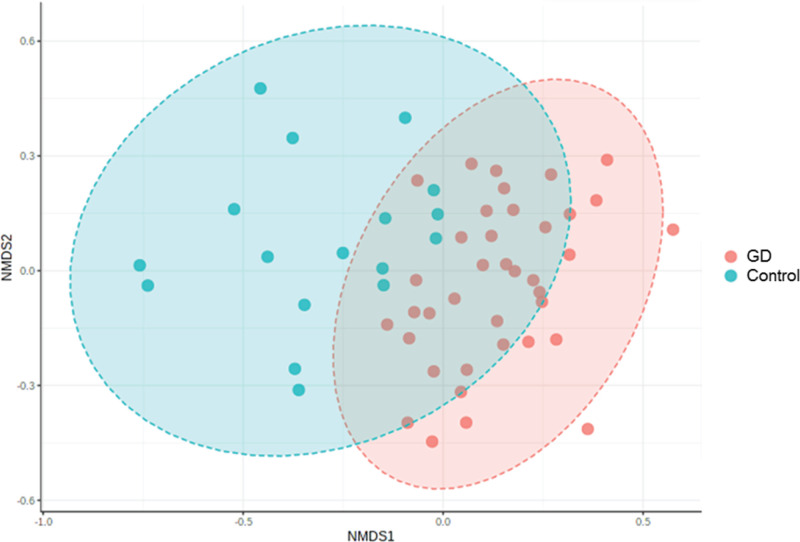
The NMDS analysis of GD patients and healthy controls Control, healthy controls; GD, GD patients.

**Figure 5:**
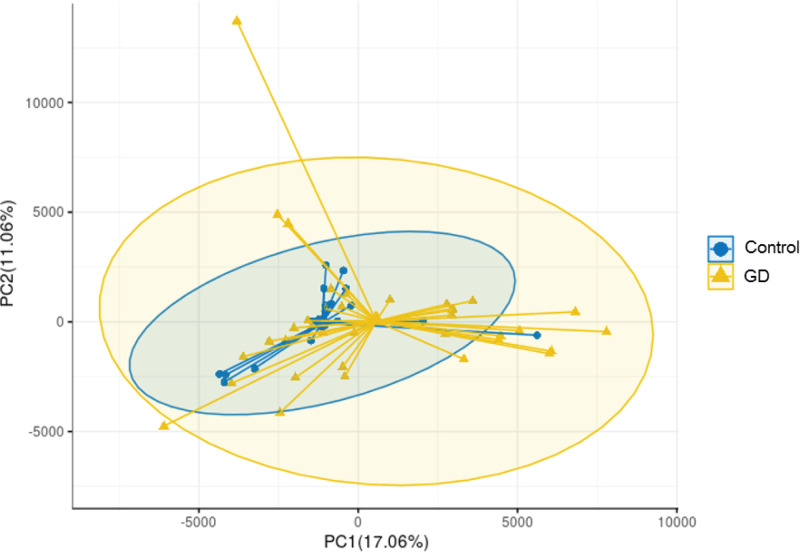
PCA plot showing microbiota diversity between GD patients and healthy controls Control, healthy controls; GD, GD patients.

Subsequently, NMDS and PCoA were performed to compare the intestinal flora diversity between the two groups. Variations in the clustering patterns were evident, suggesting differences in the intestinal flora between the two groups (Figure 3). The NMDS analysis results showed that the stress value was 0.18135, revealing a significant difference between groups (Figure 4). Further PCA showed that the composition of intestinal flora in the patients’ group was significantly different from that in the healthy control group (Figure 5).

**Figure 6:**
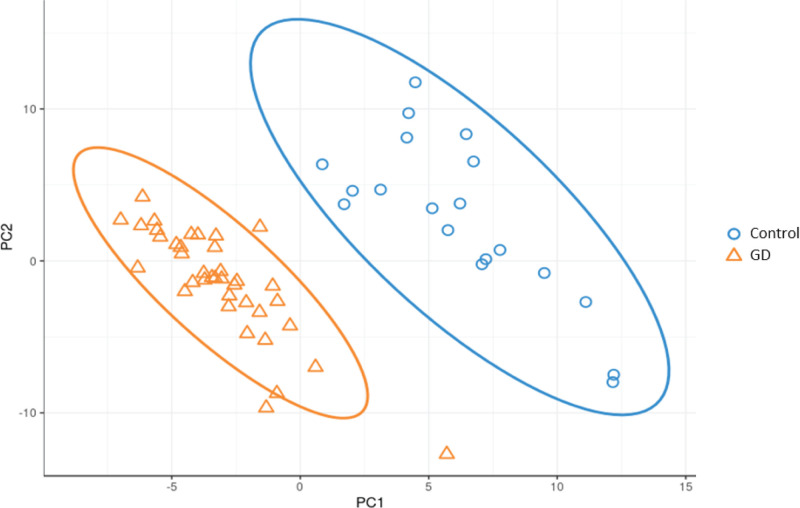
PLS-DA analysis plots of GD patients and healthy controls Control, healthy controls; GD, GD patients.

**Figure 7:**
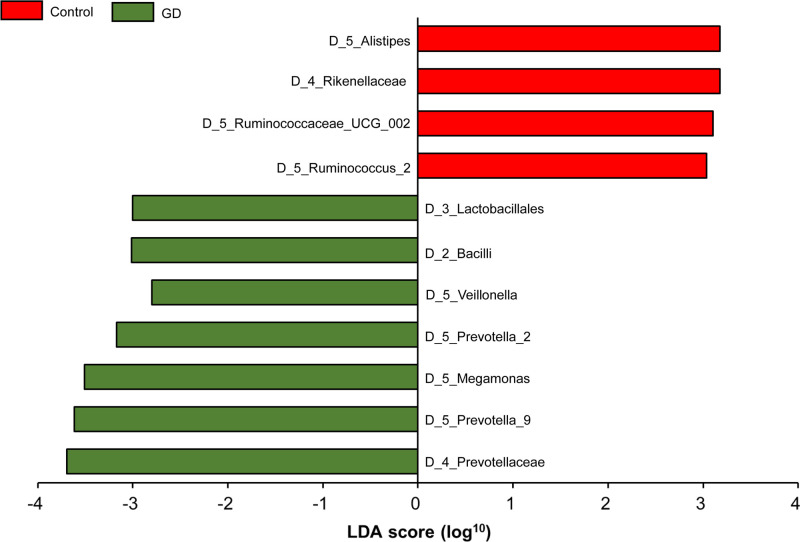
LDA score plot of GD patients and healthy controls Control, healthy controls; GD, GD patients.

**Figure 8:**
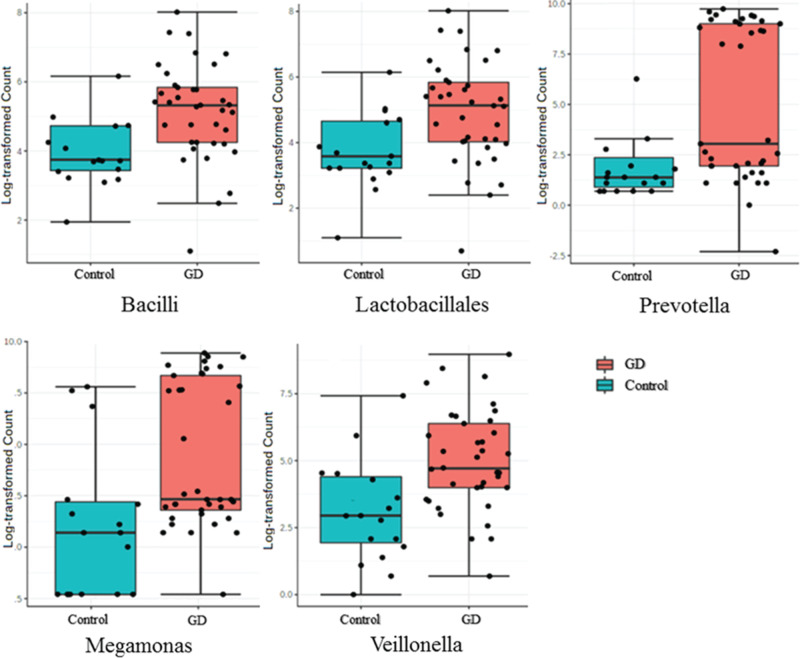
Box plot of *Bacilli, Lactobacillales, Prevotella, Megamonas*, and *Veillonella* from healthy controls (Control) and GD patients (GD)

With PLS-DA statistics, further analysis to investigate differences between the two groups were employed (Figure 6). The intestinal flora clustering of the two groups showed distinct distribution from each other, without displaying any intersections. Additional evaluation involving LEfSe for the PLS-DA analysis was conducted to determine the microbial species that exert greater influence on observed differences between the groups. The LEfSe results of significant features were listed in Table 2. The species which obtained a score higher than 3 were prioritized (Figure 7). Species unique to GD and control patients were listed in Table 3. Compared with the healthy controls, *Bacilli, Lactobacillales, Prevotella, Megamonas*, and *Veillonella* intestinal flora strains were significantly up-regulated among GD patients, as shown in the box plot (Figure 8). Moreover, among this group, there was a significant decrease in *Ruminococcus, Rikenellaceae, Alistipes* strains.

**Table 2:** LEfSe results of significant features

Species	*P*-values	FDR	GD patients	Healthy controls	LDA score
*Bacilli*	0.000651	0.013	392.84	87.90	−3.02
*Prevotella*	0.00485	0.0533	4116.40	1256.60	−3.61
*Megamonas*	0.000403	0.0399	2283.60	284.74	−3.51
*Veillonella*	0.00325	0.0533	724.38	536.68	−2.80
*Lactobacillales*	0.003	0.0659	374.14	79.90	−3.00

Abbreviation: FDR, false discovery rate.

**Table 3:** Species unique to GD and control patients (species level)

Healthy control group	GD group
*Rhizobiales*	*Enterobacteriales*
*Flavobacteriales*	*Campylobacterales*
*Clostridiales*	*Gastranaerophilales*
*Burkholderiales*	*Bacteroidales*
*Sphingobacteriia*	
*Campylobacterales*	
*Selenomonadales*	
*Coriobacteriales*	

## Discussion

In the present study, the intestinal flora changes of patients with GD are investigated by a prospective comparison with a healthy cohort. The characterization of intestinal microbiota between patients with GD and healthy individuals was analyzed using the 16S rRNA sequencing technique. It showed different intestinal flora composition and decreased diversity of intestinal microorganism for patient with GD.

Various studies also have reported an association between thyroid diseases and gut microbiota. Lauritano et al. [20] reveal overt hypothyroidism was associated with bacterial overgrowth. Zhao et al. [21] find differences in the gut microbiota composition between patients with HT and healthy controls. Some small sample research reports exact occurrence of gut microbial disturbance in patients with GD [22]. Similar to previous studies, our results indicated that patients with GD may experience different dysbacteriosis of intestinal microbiota. It is expected the present study would lay a foundation for further studies on molecular mechanism of the interaction between GD and intestinal flora, and provide a clinical reference for the diagnosis and treatment strategy of GD.

When compared with the healthy control group, the intestinal flora diversity of patients with GD was significantly reduced. This phenomenon has also been reported in various immunological diseases. Knip et al. [23] proposed that preclinical T1DM predisposes to high Bacteroidetes *to* Firmicutes ratio, a scenario that can reduce bacterial diversity and weaken community stability. Some researchers also found microbial composition and diversity in patients with inflammatory bowel syndrome (IBS) were altered when compared with those healthy controls [24]. These studies suggest that intestinal microbiota diversity is significantly affected by immune diseases.

The analysis of α and β diversity was further carried out on the two groups. The results showed that intestinal microbiota from the GD patients and healthy controls were clearly separated from each other, implying significant differences in the intestinal flora between the two groups. Jiru et al. [22] compared the intestinal microflora of 27 GD patients and 11 healthy controls, and discovered that the α diversity estimated by ACE and Chao1 was obviously elevated in healthy controls when compared with patients with GD (*P*<0.05), which was consistent with the results in our study. All these evidences accurately verified that patients with GD have different degree of changes or disorders in intestinal flora, and simultaneously involved the intestinal flora changes of α and β diversity.

It was evident that the levels of abundance of many genera of microorganisms were changed in patients GD according to the LEfSe analysis. In our result, the populations of *Bacilli, Lactobacillales, Prevotella, Megamonas*, and *Veillonella* were significantly up-regulated in the patients with GD. Similarly, a significant raise of *Prevotella* (*P*<0.033) and *Haemophilus* (*P*<0.048) in GD patients was observed in the previous study [22]. It has been reported that the increased abundance of *Prevotella* may affect the efficacy of drug therapy. For example, patients suffering from cystic fibrosis who had higher abundance of *Prevotella* in lung microbiome were less likely to respond to inhaled aztreonam therapy [25,26]. We predicted that the increased abundance of *Prevotella* may also affect the efficacy of drug therapy for GD.

A study found that *Megamonas* was decreased significantly in patients with Bechet’s disease (BD) when compared with normal individuals, and this alteration may have an association with the immune aberration in patients with BD [27]. On the other hand, *Veillonella* is a symbiotic bacterium in humans and can develop into a conditional pathogenic bacterium under certain conditions. Researchers found that *Veillonella* was enriched in primary sclerosing cholangitis patients [28], and that it was associated with the development of immune system in infants [29]. Other side, the abundance of *Ruminococcus, Rikenellaceae, Alistipes* were significantly decreased in our results. Previous article confirmed that the level of *Alistipes* in GD decreased compared with normal persons (*P*<0.025) [22]. *Ruminococcus* is a very important intestinal bacterium whose diminished levels are closely linked to several diseases such as psoriatic arthritis and inflammatory bowel disorder (IBD) [30]. Similarly, declining levels of *Rikenellaceae* also signify various metabolic and immune diseases such as Crohn’s disease [31]. In a study involving mice with diet-induced obesity, oral probiotics were found to restore the abundance of *Rikenellaceae*, improve adiposity, insulin resistance, and dyslipidemia [32]. These studies suggest an indelible association between changes in microflora abundance and immune system diseases. Whether these immune diseases and the altered flora composition will be found to be a real fuse or the two factors are causal to each other? This urgently needs to be further verified by large sample multicenter studies in the future.

It is reviewed by Jose et al. [33] that the abundance of the intestinal microbes, *Firmicutes* and *Bacteroidetes*, is associated with increased blood pressure in several models of hypertension, including the spontaneously hypertensive. Decreasing gut microbiota by antibiotics can increase or decrease blood pressure that is influenced by genotype. Li et al. [34] found dramatically decreased microbial richness and diversity, reduced bacteria associated with healthy status and disease-linked microbial function in both pre-hypertensive and hypertensive populations, when compared with the healthy controls. It describes a novel causal role of aberrant gut microbiota in contributing to the pathogenesis of hypertension. Yan et al. [35] indicated higher membrane transport, lipopolysaccharide biosynthesis and steroid degradation in hypertensive gut microbiome group, while higher metabolism of amino acid, cofactors, and vitamins in controls. These findings show specific alterations in microbial diversity, genes, species, and functions of hypertensive gut microbiome. Under the influence of these and similar molecular metabolic mechanisms, patients with hypertension and hyperlipidemia show significant intestinal flora disorder [36]. It is worth noting that all these previous studies often seem to be inclined to demonstrate the pathogenesis mechanism of intestinal flora leading to hypertension. Three patients in our study had a history of hypertension and medication history, who may be associated with genomic changes of intestinal flora that a certain extent related to hypertension. However, these three patients were not considered to have a significant impact on the overall conclusion of the present study, because their proportion in the total number of included patients was too small. Therefore, we did not further discuss the mechanism of intestinal microbial genomic changes in hypertension caused by GD.

Overall, the changes in the composition and abundance of microorganisms indicate that intestinal microbiota in patients with GD is closely related to the occurrence of hyperthyroidism diseases. Therefore, the up- or down-regulation of these strains may be an important indicator of the occurrence of GD. The molecular mechanism of the relationship between GD and intestinal bacterial changes will be studied in the further. And the changes in metabolome of intestinal bacterial from GD patients will also be further studied.

## Conclusion

By comparing the intestinal flora of GD and healthy controls, it is found that the diversity of microbial strains is significantly reduced in GD patients, and patients with GD will undergo significant changes in intestinal microbiota. These conclusions are expected to provide a preliminary reference for further researches on the interaction mechanism between intestinal flora and GD.

## Limitations

In our study, (a) 16S rRNA analysis was used to identify the genus of microorganisms, however, it cannot be used to further identify the species level of the microorganisms. (b) Relevant research showed that different species of microorganisms in the same genus might have completely opposite effects on human health. Our cross-sectional study cannot provide the causal relationship due to the gut microbiota had the time varying issue. (c) The conclusion in context of Chinese dietary habits may not be fully applicable to the European-American populations, due to the fact that the Chinese diet is generally lighter, and all participants in the present study were told to maintain a light diet for 1 week prior to fecal sampling. Therefore, the present results can only provide a preliminary reference for the relationship between intestinal microorganisms and GD.

## Abbreviations

ACE: abundance-based coverage estimator
ANOSIM: analysis of similarities
BD: Bechet’s disease
GD: Graves’ disease
HT: Hashimoto’s thyroiditis
LDA: linear discriminant analysis
LEfSe: LDA effect size
NMDS: non-metric multidimensional scaling
OTU: operational taxonomic unit
PCA: principal component analysis
PCoA: principal coordinate analysis
PLS-DA: Partial Least Squares Discriminant Analysis
SPSS: Statistical Package for the Social Sciences
TRAb: TSH receptor antibody
TSH: thyroid stimulating hormone
T1DM: type 1 diabetes
